# Giant solitary primary intracranial lymphoma masquerading as meningioma: a case and review of literature

**DOI:** 10.11604/pamj.2017.28.196.13996

**Published:** 2017-11-01

**Authors:** Junhong Li, Chuanfen Lei, Seidu A. Richard, Yanhui Liu

**Affiliations:** 1 Department of Neurosurgery, West China Hospital, Sichuan University, 37 Guo Xue Xiang Road, Chengdu, 610041, PR, China; 2 Department of Pathology, West China Hospital, Sichuan University, 37 Guo Xue Xiang Road, Chengdu, 610041, PR, China; 3 Department of Immunology, Jiangsu University, 301 Xuefu Road, Zhenjiang, Jiangsu, 212013 PR, China; 4 Department of Surgery, Volta Regional Hospital, PO, Box MA-374, Ho, Ghana, West Africa

**Keywords:** Lymphomas, meningioma, immunocompetent, intracranial, parenchyma

## Abstract

Non-Hodgkin's lymphomas (NHL) with intracranial origin are very rare and constitutes about 1-2% of primary central nervous system lymphomas (PCNSL). Diffuse large B cell lymphoma (DLBCL) is the most common subtype of NHL and mostly seen in immunocompromised patients. Therefore, the occurrence of giant solitary DLBCL in an immunocompetent patient is puzzling. We present a case of 68-year-old man who was admitted at our facility with a history of “hypomnesia of two (2) months” duration. Magnetic resonance imaging (MRI) revealed a space occupying lesion in the bilateral frontal lobe and corpus callosum measuring about 5.4cm * 4.6cm * 3.8cm with mixed signal intensities and vasogenic edema around the mass. Radiological, this mass was mistaken for meningioma until histopathological studies revealed DLBCL. Giant solitary primary intracranial lymphomas are very rare and can be mistake for meningioma even with very experience radiologist or neurosurgeon since the radiological features of PCNSL can be very unspecific. We achieved to total resection because of the giant and solitary nature of our case. The prognosis of PCNSL is general very poor when the patient is immunocompromised. In immunocompetent patients, who are well managed with surgery and chemotherapy, the overall survival and quality of life can very encouraging.

## Introduction

Primary central nervous system lymphoma (PCNSL) constitutes about 1-2% of all Non-Hodgkin's lymphomas (NHL) [1]. Diffuse large B cell lymphoma (DLBCL) is the most prevalent subtype, constituting about 90-95% of the total number of lymphomas. DLBCL are often observed in immunocompromised patients as assertive intraparenchymal lesions [2-4]. Also, NHL comprises of about 2-7% of primary intracranial tumors [1, 2, 5]. PCNSL mostly arises from the brain parenchyma although spinal cord, eyeball and cranial nerve have been reported in literature [4, 6, 7]. The brain parenchyma lymphomas are associated with diffuse edema as well as increased intracranial pressure (ICP), mental and personality changes [4, 8]. Computer tomographic scan (CT-Scan) and magnetic resonance imaging (MRI) usually show a single or multiple extra-axial dura centered lesions, which enhance diffusely on contrast enhancing studies [7]. Although the gold standard treatment is surgical excision of the lesions, total resection is usually not achievable because of multifariousness of the masses or their infiltrative nature. Radiotherapeutic treatment modality is also very crucial in the management of PCNSL although some author have observed challenges with some lymphoma subtypes especially dural follicular lymphomas [7]. Furthermore, some authors have indicated that chemotherapy is of indeterminate significance in patients with PCNSL [7]. We present a case of giant solitary primary intracranial lymphoma with cerebral falx as its base masquerading as meningioma.

## Patient and observation

**Figure 1:**
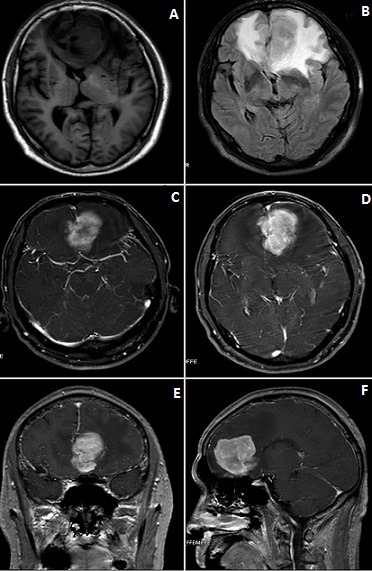
(A-F) are MRI showing the lesion: the lesion is located at bilateral frontal lobe and corpus callosum with massive vasogenic edema surrounding it

We present a case of 68-year-old man who was admitted at our facility with a history of “hypomnesia of two (2) months” duration. He denied headaches, vomiting, visual loss and palpitations. He however has associated urine and stool incontinence which started two (2) weeks prior to his illness. He was diagnosed hypertensive 10 years ago and has been on antihypensives ever since the diagnosis was made. General physical examination was unremarkable with no palpable lymph nodes and no signs of chronic anemia. All systems were grossly normal. Neurological examination did not yield much. Cranial nerves were normal. Ophthalogical examination was unremarkable. Digital rectal examination revealed a weak sphincter tone. Preoperative laboratory investigations revealed grossly normal complete blood count (CBC) with slightly increase in the percentage of neutrophilic segmented granulocyte in the peripheral blood as well as a decrease in the percentage of lymphocytes. Liver enzymes where markedly deranged. All other routine laboratory investigations were normal. MRI revealed a space occupying lesion in the bilateral frontal lobe and corpus callosum measuring about 5.4cm * 4.6cm * 3.8cm with mixed signal intensities (Figure 1). The lesion was hypo-intense on T1 and hyper-intense on T2. The mass enhanced markedly in enhancement scan, with evident edema zone in the surrounding area. FLAIR sequences also showed a hyper-intense lesion. The frontal horn of lateral ventricle was compressed and translocated, no obvious abnormality was found in the skull bone. A working diagnosis of meningioma with cerebral falx as its base was made to rule our other lesions like lymphoma, dural metastasis, solitary fibrous tumour or leiomyosarcoma. CT scan as well as MRI of the chest, abdomen and pelvis did not yet much.

**Figure 2:**
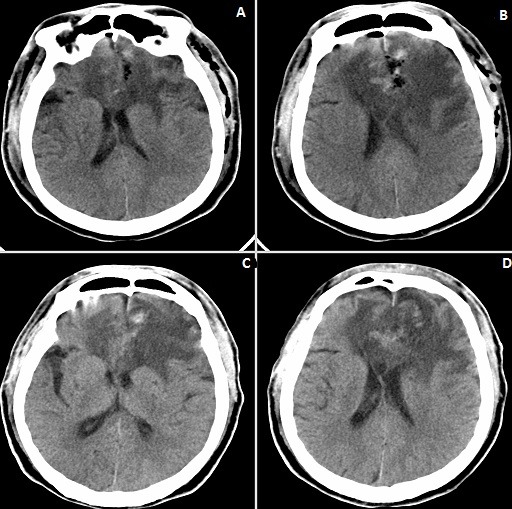
(A-D) are postoperative CT-Scans: A & B were take on postoperative day one (1) while C & D were taken on postoperative day six (6)

**Figure 3:**
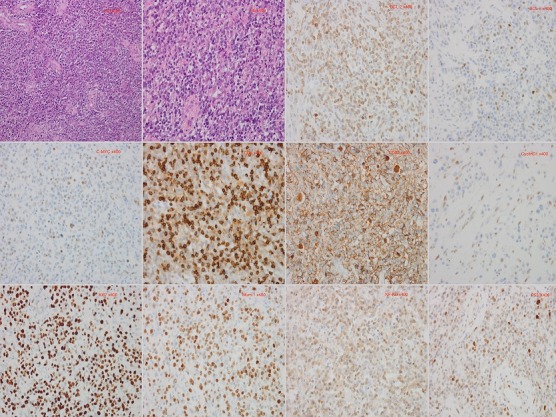
Are immunohistochemical staining of the lesion: HE x200, HE x400, BCL-2 x400, BCL-6 x400, CD5 x400, C-MYC x400, CD20 x400, CyclinD1 x400, Ki67 x400, Mum-1 x400, NF-KB x400, P53 x400

We use the left frontal craniotomy via the anterior skull base to access this giant lesion. Intraoperatively, we observed that the scalp and skull were normal. After drilling and removing the bone flat, the dura was opened. We observed that the ICP was high and CSF was decompressed to decrease the ICP. The lesion was seen occupying the left frontal lobe and extending down to the anterior skull base as well as the anterior portion of the corpus callosum. There was marked edema around the lesion as well as shifting of falx toward the right frontal lobe. The lesion had a slightly tough texture with general blood supply coming from the anterior cerebral artery and its branches. There was significate adhering of the lesion to the brain tissue or matter. We attained total resection of the lesion. Postoperative CT-Scan done on day one (1) and day six (6) after surgery confirmed total resection of lesion (Figure 2). Pathological examination showed lymphoid tissue with hyperplastic and diffuse proliferation of medium-sized lymphoid cells. Immunohistochemical results showed that: CD20 (+), CD3p (-), CD10 (-), bcl-6 (+), Mum-1 (+), Cyclin D1 (+), CD30 (-), CD5 (+), P53 (+), C-MYC (+), NF-KB (+), bcl-2 (+), Ki67 (+), situ hybridization EBER1/2 (-). PCR and capillary electrophoresis gene scanning revealed the IgΚ gene clonal amplification peaks (Figure 3). Pathological diagnosis of non-Hodgkin's lymphoma, subtype; diffused large B cell lymphoma (WHO 2008, CD5+, high value-added activity) was made. Bone marrow examination also confirmed non-Hodgkin's lymphoma. The patient recovered quickly after operation with no neurological deficits. He was discharged home ten (10) days after operation. Chemotherapy was initiated three (3) month after the operation by which time he had recovered fully from the surgical operation. One year follow-up revealed massive improvement of his life. The ethical committee of the hospital full approved our case study. The patient and his relatives were informed about our intension to involve him in a case study and he/they agreed to partake in the study. He/they signed the concern form before the operation was carried out according to all surgical protocols.

## Discussion

PCNSL constitutes about 1-2% of all NHL [1]. DLBCL is the most prevalent subtype, constituting about 90-95% of the total number of lymphomas. DLBCL are often observed in immunocompromised patients as assertive intraparenchymal lesions [2, 3]. Our patient was not immunocompromised so falls within the remaining 5-10% of DLBCL patients who present with intraparenchymal lesions. Also, NHL comprises of about 2-7% of primary intracranial tumors [1, 2, 5]. PCNSL mostly arises from the brain parenchyma although spinal cord, eyeball and cranial nerve have been reported in literature [6, 7]. PCNSL are commonly seen in middle aged group with average age at 60 years [4]. The female to male ratio is 4:1 [7] Studies have proven that DLBCL originates from mature B-cells at distinctive stages of differentiation. Several gene mutations facilitate transformations in B-cells, altering the gene secretion and facilitating a carcinogenic alteration [4, 9]. Our case is much interesting because of the giant and solitary nature of the lesion which initially can be mistaken as meningioma even with very experienced radiologist or neurosurgeon. Furthermore, a very diligent literature search in Medline as well as universal search engine (google scholar) yielded no images describing a solitary giant intracranial of CNS lymphoma like our case. The clinical manifestations of PCNSL comprises of neurological disorders and neuropsychiatric disorders. While neurological disorders are seen in about 80% of patients, neuropsychiatric disorders are seen in about 20-30%. High ICP is manifested in almost all patients [4, 8]. Furthermore, neurological signs and symptoms characteristically rely on the site of the lesion. The typical manifestations are headache, seizures, focal neurological deficits and visual disorders. Other unusual manifestations comprise of nausea, vomiting, ataxia, cranial nerve dysfunction and infrequently bilateral visual and hearing loss [7, 10]. CT-Scan and MRI usually show a single or multiple extra-axial dura centered lesions, which enhance diffusely on contrast enhancing studies [7]. In some patients, the lesion may be seen on radiology as en-plaque thickening of meninges, dural tail sign, parenchymal vasogenic edema, invasion of underlying brain or superimposing calvaria [7, 11]. Many authors have confirmed that dural-based mucosal-associated lymphoid tissue lymphomas are extremely analogous to meningiomas on imaging [2, 6, 12, 13]. On MRI, the appearance of PCNSL is inconstant. On T1, the lesions vary from iso to hyper-intense. On T2, the lesions often vary from iso to hypo-intense.

Gadolinium enhancement often show lesions with varying intensities and homogeneous patterns [2, 14, 15]. Giant lesions may initiate peritrigonal edema and subependymal venous engorgement [2, 15]. Interestingly, the MRI of patients show a lesion that was hypo-intense on T1 and hyper-intense on T2 (Figure 1). Furthermore, the most significant working diagnosis in our patient is meningioma since location with peritumuor edema also favors meningioma more the lymphoma. The male gender in our case also makes it more puzzling and will convince any radiologist that it is meningioma since lymphomas rarely occur in males (M: F = 1:4). Nevertheless, numerous likenesses exist between these two lesions, both are common at his age and frequently involve more than one extraaxial sites. Moreover, on radiological imaging these two disorders are seen as diffuse enhancing extra-axial lesions. Conversely, vasogenic edema and the lack of calvarial hyperostosis is mostly seen in primary dural lymphomas [7, 11].

Additional rare radiological differentials are; dural metastasis, solitary fibrous tumour, leiomyosarcoma, plasmacytoma, inflammatory pseudotumour, neurosarcoidosis, Castelman's disease and rheumatoid nodule [7,11]. The definitive diagnosis often relies on histopathology as well as immuno-phenotyping since radiological features are similar. Furthermore, CT scan of the chest, abdomen and pelvis and bone marrow examination is highly recommended in situation where the radiological diagnosis is not clear cut. Positive findings will shift the diagnosis towards PCNSL more than meningioma. Additionally, MRI of the brain and spine is obligatory for clinical staging of the lymphoma [7].

Although the gold standard treatment is surgical excision of the lesions, total resection is usually not achievable because of multifariousness of the masses or their infiltrative nature [7]. On the other hand, some authors are of the view that surgery does not benefit patients with PCNSL. Therefore, they kick against surgery with the explanation that surgery increase neurological deficits [16]. Looking at the size of the lesion in our patient, we think the only reliable remedy is surgery because surgical resection of the lesion will decrease ICP as well as relief any impeding brain herniation. The basic principle of PCNSLs consolidation therapy is to clear the remaining lymphocytes and reduce the risk of recurrence. Chemotherapy comprise of drugs that can cross the blood-brain barrier, such as methotrexate, corticosteroids, rituximab and so on [1]. Methotrexate is currently used in combination with other chemotherapeutic drugs such as cytarabine, alkylating agents, nitrosoureas, and temozolomide etc to prolong overall survival. High dose methotrexate (HD-MTX) based chemotherapy is often the first-line treatment and the first choice for patients with PCNSLs [1]. There is still lack of clinical evidence on the use of intrathecal or intraventricular methotrexate injection. Also, as to whether radiotherapy, especially whole brain radiotherapy (WBRT), is beneficial to the patient also remains controversy [1, 16]. Several authors support radiation dose comprising of WBRT and booster, ranging from 45-60 Gy [13, 17-19]. On the other hand, G-PCNSL-SG-1 trial does not recommend the use of consolidated WBRT as first-line therapy. Studies have proven that WBRT does not increase the overall survival of patients but rather reduce the quality of life as well as lead to neurocognitive hypofunction [20]. A combination of chemotherapeutic drugs and autologous stem cell transplantation (HCT-ASCT) has also proven to lessen disease load as well as augment overall survival [21]. Microscopically the DLBCL is composed of varying proportions of hyperplastic and diffuse proliferation of medium-sized lymphoid cells. Occasionally, follicular centre B cells, mixtures of small cleaved centrocytes and large non-cleaved centroblasts that have at least in part, a follicular growth pattern are also seen in follicular B cell lymphomas [7]. Immunophenotype using flow cytometry and/or immunoperoxidase stain is imperative to extra categorize low-grade B-cell lymphomas. These lymphomas are typically positive for pan-B cell markers, such as CD19, CD20 and CD22. DLBCL often have nodular growth pattern and are typically CD10, bcl2 positive and CD5, CD23 negative [7, 22, 23]. Amen et al confirmed better overall survival and disease-free survival in DLBCL with no BCL2 and Cyclin D2 secretion [4, 24]. Nevertheless, other authors have demonstrated a negative effect of the secretion of BCL2 only in the subgroup of activated B-cell DLBCL [4, 25]. Modifications in BCL6 protein secretion may lead to failure in cell differentiation and continuous stimulation as well as proliferation. Subsequently, it facilities a better cell survival and genetic instability that partake in carcinogenic transformations [4, 26, 27]. Patients with lack or mutated p53 are often seen with more assertive disease and poorer outcomes [4, 28]. A few authors are of the view that p53 may be deactivated by the BCL6 gene during the genesis of lymphoma [4, 26, 27]. Studies have also indicated that MYC is linked with poorer total remission as well as worse overall survival rates [4].

## Conclusion

Giant solitary primary intracranial lymphomas are very rare and can be mistake for meningioma even with very experience radiologist or neurosurgeon since the radiological features of PCNSL can be very unspecific. The gold standard treatment is surgical excision of the lesions, although total resection is usually not achievable because of multifariousness of the masses or their infiltrative nature. We achieved to total resection because of the giant and solitary nature of our case. The prognosis of PCNSL is general very poor when the patient is immunocompromised. In immunocompetent patients, who are well managed with surgery and chemotherapy, the overall survival and quality of life can very encouraging.

## Competing interests

The authors declare no competing interest.
